# Conservative Treatment of Bisphosphonate-Related Osteonecrosis of the Jaw in Multiple Myeloma Patients

**DOI:** 10.1155/2014/427273

**Published:** 2014-06-17

**Authors:** Pelagia I. Melea, Ioannis Melakopoulos, Efstathios Kastritis, Christina Tesseromatis, Vasileios Margaritis, Meletios A. Dimopoulos, Evangelos Terpos

**Affiliations:** ^1^Department of Clinical Therapeutics, National and Kapodistrian University of Athens, School of Medicine, Alexandra General Hospital, 80 Vas. Sofias Avenue, 11528 Athens, Greece; ^2^Department of Oral and Maxillofacial Surgery, Ygeia Hospital, Athens, Greece; ^3^Department of Pharmacology, Faculty of Medicine, National and Kapodistrian University of Athens, Athens, Greece; ^4^Department of Ph.D. Program in Public Health, Faculty of Health Sciences, Walden University, MN 55401, USA

## Abstract

The use of intravenous bisphosphonates (pamidronate or zoledronic acid) is the cornerstone for the management of multiple myeloma-(MM-) related bone disease. However, osteonecrosis of the jaw (ONJ) is a rare, but sometimes difficult to manage, adverse effect of bisphosphonates therapy. A retrospective review of all MM patients who were treated with bisphosphonates in our department, from 2003 to 2013, and developed ONJ was performed. According to inclusion criteria, 38 patients were studied. All these patients were treated as conservatively as possible according to the American Association of Oral and Maxillofacial Surgeons criteria. Patients were managed with observation, oral antibacterial mouth rinse with chlorhexidine, oral antibiotics, pain control with analgesics, nonsurgical sequestrectomy with or without simultaneous administration of antibiotics, or major surgery with or without antibiotics. Healing of the lesions was achieved in 23 (60%) patients who were treated with conservative measures; the median time to healing was 12 months (95% CI: 4–21). The number of bisphosphonates infusions influenced the time to healing: the median time to healing for patients who received <16 infusions was 7 months and for those with >16 infusions was it 14 months (*P* = 0.017). We conclude that a primarily nonsurgical approach appears to be a successful management strategy for bisphosphonate-related ONJ.

## 1. Introduction

Bisphosphonate-related osteonecrosis of the jaws (BRONJ) is an avascular osteonecrosis of the jaws, associated mainly with intravenous administrated bisphosphonates but also with oral bisphosphonates. Intravenous bisphosphonates are used for the management of bone disease and bone metastases, caused by multiple myeloma and other solid tumors, for example, breast cancer, prostate cancer, and lung cancer [1, 2]. BPs main action is to inhibit osteoclast function and subsequent bone resorption, resulting in the prevention of loss of bone mass and skeletal related events, such as pathologic fractures and pain, caused by the underlying disease [3, 4]. A great number of patients with cancer benefit from the therapeutic results of BPs. Nevertheless bisphosphonate-related osteonecrosis of the jaw (BRONJ) has been described as an adverse effect of these drugs in various malignancies [5–8], with negative effect on the quality of life of the patients [9].

The diagnosis of osteonecrosis is clinical and according to suggested criteria [10] requires the presence of exposed bone in the jaw area for more than eight weeks, in a patient under current or previous treatment with a bisphosphonate, with no history of radiation therapy to the head and/or neck area.

The incidence of BRONJ ranges considerably due to various factors, such as type of bisphosphonate, type of cancer, way of administration, time of exposure, and number of infusions [11–14]. The risk of developing BRONJ in multiple myeloma patients receiving intravenous zoledronic acid or pamidronate is relatively high. Previous studies from our team as well as from other groups have identified tooth extraction or chronic trauma of the oral mucosa caused by poorly fitting dentures, poor oral hygiene, and number and duration of zoledronic acid administration as the main triggering factors for the development of ONJ [12, 15–18]. However, spontaneous development of BRONJ is also possible and has been reported [12, 17].

Several approaches have been evaluated for the treatment of patients who developed BRONJ and many management strategies have been proposed. Nevertheless, it seems that taking preventative measures is the most effective way to face BRONJ. In our current study we report on the outcome of our series of MM patients who developed ONJ and discuss management issues.

## 2. Materials and Methods

A retrospective review of multiple myeloma patients who were diagnosed with BRONJ from July 2003 until September 2013 and were treated in the Department of Clinical Therapeutics (Athens, Greece) was conducted. All the patients reporting symptoms and/or signs compatible with the probability of development of osteonecrosis were prospectively evaluated. BRONJ was diagnosed by a specialized maxillofacial surgeon (IM) according to the following criteria: patients, with no history of head and/or neck radiotherapy, currently or previously treated with bisphosphonates and presence of exposed bone in the maxilla and/or the mandible for more than eight weeks. All cases with denosumab associated necrosis were excluded, as well as cases in which the whole treatment was not performed by the same group, to avoid data that was not confirmed.

From 105 patients with osteonecrosis of the jaws under treatment with antiresorptive agents for any reason (solid tumor metastasis, multiple myeloma, etc.), thirty eight patients were selected according to the aforementioned criteria, that is, multiple myeloma patients with osteonecrosis of the jaw, caused by IV bisphosphonate therapy, who were treated in our clinic from the time of diagnosis of their disease. Biopsy was performed, if exclusion of myelomatous involvement was necessary. All species removed surgically (sequestra debridement) were also histologically evaluated.

The determination of the stage of osteonecrosis was made according to the definition and staging system published by the American Association of Oral and Maxillofacial Surgeons (AAOMS) updated position paper as follows: stage 0, no clinical evidence of necrotic bone, but nonspecific clinical findings and symptoms; stage 1, exposed and necrotic bone in patients who are asymptomatic and have no evidence of infection; stage 2, exposed and necrotic bone associated with infection as evidenced by pain and erythema in the region of the exposed bone with or without purulent drainage; and stage 3, exposed and necrotic bone in patients with pain, infection, and one or more of the following: exposed and necrotic bone extending beyond the region of alveolar bone (i.e., inferior border and ramus in the mandible, maxillary sinus, and zygoma in the maxilla) resulting in pathologic fracture, extra-oral fistula, oral-antral/oral-nasal communication, or osteolysis extending to the inferior border of the mandible or sinus floor.

A standardized and comprehensive history was obtained from each patient at the initial consultation. Data was abstracted, using a standardized template that collected patient information, medical history, and dental history, including recent dental extractions. Information concerning myeloma treatment, for example, number of infusions, duration of BP exposure, time for healing, and time of death, was also evaluated. All patients underwent comprehensive clinical evaluation and panoramic and/or intraoral periapical radiographs, when a com beam CT scan was performed in some cases. Management was provided according to general guidelines designed to minimize symptoms and/or achieve resolution of lesions.

The protocol we have followed since 2003 for all patients diagnosed with BRONJ was established based on the data of bibliography and the observation and personal experience of the attendant maxillofacial surgeon (IM). According to our protocol bisphosphonate therapy was interrupted in patients who developed BRONJ at the time of diagnosis according to guidelines [14]. Initial management in all cases was as conservative as possible. Regardless of stage, chlorhexidine rinses were prescribed for the majority of patients and mobile fragments of bone were managed with non-surgical sequestrectomy (simple removal of mobile bone fragments), typically without the need for local anesthesia. In patients with BRONJ and no signs of inflammation, avoidance of surgical dental treatment (extractions, implant therapy, and oral surgery procedures), amelioration of oral hygiene, and use of oral antiseptic mouth rinses (chlorhexidine 0.12% for 3 weeks per month, other antiseptic for 1 week per month) were recommended. Patients with artificial dentures were advised to remove them, in order to reduce the contact of the denture with the exposed bone and avoid further trauma of the mucosa. When inflammation was present, antimicrobial chemotherapy was given, usually metronidazole 500 mg twice a day for 2 weeks or aminopenicillins in combination with metronidazole for 15 days in more severe cases. Alternative choice for patients allergic to aminopenicillin was moxifloxacin for 10 days, as post antibiotic effect makes this treatment equal to a 15-day therapy. According to literature, the use of clindamycin in patients with BRONJ is not indicated after 2005 [12]. When bone spindles were present, only minor debridement procedures were attempted, in order to reduce trauma of the adjacent soft tissues. Observation and/or minor debridement procedures were also attempted, in case of spontaneous apoptosis of sequestra. When radiographic appearance of a sequestrum was observed, minor surgical sequestrectomy under local anesthesia and antibiotic treatment was attempted. Patients at stage 3 or patients who showed recurrence were treated with major surgical intervention, that is, peripheral ostectomy under general anaesthesia and antibiotic therapy.

Absence of exposed necrotic bone, absence of any signs of inflammation of the soft tissues, complete healing of the mucosa, and absence of subjective complains about pain and/or numbness for more than 3 months were considered as complete healing criteria.

## 3. Results

A total of thirty eight multiple myeloma patients were diagnosed with BRONJ, 25 males (66%) and 13 females (34%). The patients' age at time of BRONJ diagnosis ranged from 29 to 83 years, with mean age of 66 years. Twenty-six patients developed BRONJ in the mandible, 11 in the maxilla, and one patient in both mandible and maxilla. Thirty-three patients (87%) were treated with zoledronic acid (Zometa; Novartis Pharmaceuticals Corporation, East Hanover, NJ, USA) of 4 mg infused over 15 minutes every 4 weeks, 1 patient with pamidronate (Aredia; Novartis Pharmaceuticals Corporation, East Hanover, NJ, USA), of 90 mg infused every 4 weeks, and 4 patients (11%) were treated with both zoledronic acid and pamidronate. Mean number of BP infusions was 25.5 (6–83). The triggering factor of BRONJ development was oral surgery, such as tooth extraction in 22 cases, chronic mucosa trauma from artificial dentures in 5 cases, periodontal and/or periapical inflammation in 4 cases. Seven cases developed spontaneously, six of them at the mylohyoid ridge (Table 1).

Biopsy and histological assessment of the sequestra were performed in 29 cases, which confirmed the complication. Three patients (8%) were diagnosed with stage 0, eight patients (21%) with stage 1, seventeen cases (45%) with stage 2, and ten (26%) with stage 3 ONJ (Table 2).

Three patients were treated only with observation, mouth rinses with chlorhexidine 0.12% for 3 weeks per month, other antiseptic for 1 week per month, in order to avoid disturbance of the oral flora, and removal of the bony edges of the lesion. One showed complete healing, one remained stable, without any signs of inflammation or pain until death, and one patient developed higher stage of ONJ (stage 2) and was treated with antibiotics. Ten patients were treated with chlorhexidine 0.12% for 3 weeks per month, other antiseptic for 1 week per month, and antibiotics, whenever inflammation appeared. Eight of these patients remained stable for a mean follow-up of 24 months (3–48), one was completely healed after 8 months, with a 5 months follow-up after healing and one patient developed a higher stage of ONJ and is scheduled for surgery, whenever his health status permits. Seven patients had spontaneous apoptosis of sequestra and they all showed complete healing. Mean follow-up was 27 months (8–40) after the confirmation of healing. No recurrence was observed in any of these patients, until the last-follow up or until death. Conservative sequestrectomy was attempted after a meantime of 12 months under antibiotic therapy in 16 cases. Eleven of these cases showed complete healing; one case was not yet completely healed at the time of the last follow-up, one patient died during the follow-up after healing period, and three cases underwent a second minor surgery before achieving complete healing. Major surgical intervention was attempted in 2 patients with stage 3 BRONJ. Complete healing was observed in both cases, although one patient underwent a second surgery after a period of 5 months, in order to reverse the failure of the first surgery. The other patient underwent two surgeries in different locations each time—one in the maxilla and one in the mandible, since he had developed ONJ bilateral in the maxilla and the mandible. Mean follow-up after healing in both cases was more than 6 months (Table 3).

**Figure 1:**
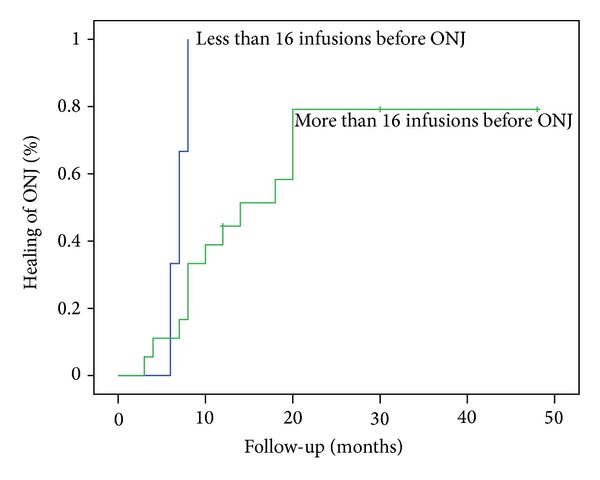
Median time to healing in association with the number of infusions of bisphosphonates.

In patients where healing was stated (*N* = 24, 63%), by removal of bony edges, spontaneous apoptosis of the sequestra, or sequestrectomy, the median time to healing was 12 months (95% CI 4–21). A statistically significant difference (*P* = 0.017) was found between groups with more and less than 16 infusions of bisphosphonates, when median time to healing for those with <16 infusions was 7 months and median time to healing for those with >16 infusions was 14 months (*P* = 0.017; Figure 1).

## 4. Discussion

The incidence of BRONJ is yet undetermined. According to many studies of patients with multiple myeloma, breast, or prostate cancer, who received intravenous amino-BP therapy, the occurrence of osteonecrosis is estimated to be approximately 4–11% [7, 13, 18]. In our study which included only multiple myeloma patients, the incidence of ONJ was almost 6%. The probability of developing BRONJ ranges due to various risk factors. The number and frequency of infusions, but mainly the cumulative dose of BP, are strongly associated with the risk of BRONJ [12, 19]. Invasive dental procedures, that is, tooth extractions, implant therapy, oral surgery, as well as mucosa trauma by poor fitting dentures have been reported as the most important triggering factors of developing this complication. However, spontaneous development of BRONJ occurs in approximately 20% of the patients who develop BRONJ [20, 21]. Indeed, in our study 57.9% of the patients who developed osteonecrosis underwent dental extraction, 13.2% had chronic mucosa trauma by artificial dentures, 10.5% of ONJ patients developed ONJ due to periodontal and/or periapical inflammation, and in 18,4% patients it occurred spontaneously, which comes in agreement with the latest reviews. The mean number of infusions was 25.5 and the mean time of BP exposure was 36.5 months. In the present study, lesions occurred more frequently in the mandible than in the maxilla (67% versus 33%). This ratio is also confirmed by several studies [22–24].

The management of BRONJ is a difficult goal to achieve and still remains controversial, since consensus standard protocol has not yet been established. According to the guidelines of the AAOMS, treatment strategies of BRONJ emphasize mainly the elimination of pain and inflammation and the reduction of the exposure of the necrotic bone and secondarily they emphasize the complete healing of the lesion. Several methods have been proposed, which can be categorized as nonsurgical or conservative [25–27] and surgical approaches [28, 29].

Nonsurgical treatment includes a combination of antiseptic mouth rinses, antimicrobial chemotherapy, when inflammation occurs, and nonsurgical sequestrectomy and/or debridement. The outcomes of most studies [24–27] seem to be satisfactory. According to one of the largest—in terms of patients—retrospective study by Lerman et al., 71–80% of the cases, treated conservatively improved or remained asymptomatic and stable [25]. In our study 63% of the patients who were treated with conservative measures (removal of bony edges, spontaneous apoptosis of sequestra, or minor surgical intervention) achieved complete healing and another 23.7% remained asymptomatic and stable, while in 5.2% of the patients major surgical interpretation was performed, because of failure of the conservative treatment. Van den Wyngaert et al. suggest that there are several factors, such as stage of ONJ, patient's health condition, time of exposure to BP, type of BP therapy, use of chemotherapy before ONJ, which should be considered in order to proceed to a specific treatment of ONJ, although it seems that strictly conservative treatment at low stages of the complication can lead to healing in about half of the cases [26]. In agreement with the above results a study by Moretti et al. confirms management of pain with minimally invasive treatment in more than 60% of the cases, while all of the patients who underwent sequestrectomy—spontaneously or gently induced by the surgeon—achieved complete healing [27]. In the present study 87.5% of stage 1 patients, 59% of stage 2, and 50% of stage 3 patients were healed.

On the other hand, radical surgical treatment of ONJ, including extensive sequestrectomy and limited or extensive bone resection, has showed healing of BRONJ in several studies [29–33]. The results of the study by Wilde et al. showed that 88% of the patients, treated surgically, achieved complete healing of ONJ. Nevertheless, a statistically high failure rate in stage 3 ONJ, approximately 36%, may initiate doubts about the efficiency of the surgery, while adequate surgical planning and high degree of experience on the determination of the resection margins are clearly pointed out by the author. Stockmann et al., at a study with 80 patients, report a success rate of about 89%, which declined to 84% within 14 months postoperatively [31]. The outcomes of a review by Kühl et al. showed that, when comparing the results of conservative and surgical treatment of BRONJ, it seems that there is no difference regarding the success of treatment (e.g., 60.5% versus 60.4%), although it appeared that complete healing of BRONJ after conservative treatment is only successful in low stages of the complication [32]. We also conclude (*P* = 0.017) that the number of BP infusions is associated with the median time to healing. Patients who received less than 16 infusions achieved healing in the half time, compared with patients who received more than 16 infusions (7 versus 14 months).

Other therapeutic approaches, such as medical ozone [34] and ND:YAG laser stimulation [35, 36] have given encouraging results in the management of patients with ONJ but the experience with these methods is limited.

In the present study, major surgical intervention was decided only at high levels of ONJ or in case of failure of conservative measures. Both patients who underwent major surgery achieved complete healing. Due to bisphosphonates discontinuation, many cases (7) of spontaneous apoptosis of the sequestra have been observed. The mean time of sequestra formation was 10.2 months where the mean time for minor surgery intervention (15 patients) was 15.6 months. It could be a reasonable thought that in that period of time the bone turnover in the necrotic area starts to work. When treatment with IV bisphosphonates could be stopped, it is reasonable to treat patients conservatively until the time where sequestra formation seems to start. Therefore, in agreement with the AAOMS guidelines, we believe that the cost-benefit for patients who are already debilitated by their malignancy leans to more conservative treatment strategies of ONJ with satisfactory results and surgical intervention should be performed only in cases of failure of the above strategies.

## Figures and Tables

**Table 1:** Patients' clinical characteristics.

Patient	Gender	Age at diagnosis	BP therapy	Number of infusions	Stage of BRONJ	Triggering factor
A.G.	Male	81	ZA	12	1	Spontaneous
A.K.	Male	61	ZA and Pam	19	3	Extraction
A.E.	Female	70	ZA	25	1	Spontaneous
B.I.	Male	50	ZA	20	2	Extraction
B.A.	Male	76	ZA	11	1	Spontaneous
B.Ir.	Female	53	ZA	25	2	Extraction
B.D.	Male	65	Z.A and Pam	80	2	Extraction
B.P.	Female	43	ZA	26	3	Extraction
B.E.	Female	79	ZA	42	3	Extraction
B.S.	Male	81	ZA	32	2	Trauma from dentures
D.A.	Male	63	ZA	28	2	Extraction
D.Z.	Male	59	ZA	6	1	Extraction
D.E.	Female	72	ZA	13	0	Trauma from dentures
G.M.	Male	82	ZA	12	2	Extraction
K.K.	Male	74	ZA	17	3	Spontaneous
K.M.	Female	72	ZA	39	1	Spontaneous
K.E.	Female	68	ZA	22	3	Trauma from dentures
K.N.	Male	78	ZA	58	2	Extraction
K.P.	Male	66	ZA	17	2	Extraction
K.V.	Male	73	ZA	30	0	Periapical abscess
K.I.	Male	69	Pam	25	2	Extraction
M.T.	Female	59	ZA	31	1	Trauma from dentures
P.O.	Female	61	ZA	48	2	Extraction
P.G.	Male	81	ZA	59	1	Trauma from dentures
P.V.	Female	57	ZA	83	2	Periapical abscess
P.T.	Male	61	ZA	15	1	Spontaneous
P.M.	Female	69	ZA	21	1	Trauma from dentures
P.Ma.	Female	71	ZA	36	1	Periodontal Inflammation
P.K.	Male	59	ZA	8	3	Extraction
P.D.	Male	61	ZA and Pam	34	2	Extraction
S.E.	Male	65	Z.A	13	3	Extraction
S.D.	Male	61	Z.A	26	3	Extraction
S.K.	Male	55	Z.A	65	2	Periodontal Inflammation
S.G.	Male	80	Z.A	45	2	Spontaneous
T.P.	Male	29	ZA and Pam	38	2	Extraction
V.C.	Male	50	ZA	25	3	Extraction
X.E.	Female	67	ZA	26	0	Extraction
Z.L.	Male	72	ZA	17	3	Extraction

Total	Male: 25 female: 13	66 Years (29–83)	ZA: 33 Pam: 1 ZA + Pam: 4	25.5 (6–83)	St 0: 3 St 1: 8 St 2: 17 St 3: 10	Extraction: 22 Trauma Dentures: 5 Periodontal/periapical inflammation: 4 Spontaneous: 7

ZA: zoledronic Acid; Pam: pamidronate.

**Table 2:** Management of ONJ by stage.

Stage	*N*	CHL rinses and observation plus removal of bony edges	Antibiotics plus removal of bony edges	Spontaneous apoptosis of sequestra	Minor surg. intervention-Sequstrectomy	Major surgical intervention
0	3	0	2 (67%)	0	1 (33%)	0
1	8	1 (12.5%)	1 (12.5%)	5 (62.5%)	1 (12.5%)	0
2	17	1 (5.9%)	4 (23.5%)	1 (5.9%)	11 (64.7%)	0
3	10	1 (10%)	3 (30%)	1 (10%)	3 (30%)	2 (20%)

Total	38	3 (7.9%)	10 (26.3%)	7 (18.4%)	16 (42.1%)	2 (5.3%)

**Table 3:** Results of ONJ treatment.

Treatment	*N*	Stable	Complete healing	Regression
CHL rinses and observation plus removal of bony edges	3	1 (33.3%)	1 (33.3%)	1 (33.3%)
Antibiotics plus removal of bony edges	10	8 (80%)	1 (10%)	1 (10%)
Spontaneous apoptosis of sequestra	7	0	7 (100%)	0
Minor surg. intervention-Sequestrectomy	16	1 (6.25%)	15 (93.75%)	0
Major surgical intervention	2	0	2 (100%)	0

Total	38	10 (26.3%)	26 (68.4%)	2 (5.3%)
